# Complete Genome Sequences of Chikungunya Virus Strains Isolated in Mexico: First Detection of Imported and Autochthonous Cases

**DOI:** 10.1128/genomeA.00300-15

**Published:** 2015-05-07

**Authors:** José Alberto Díaz-Quiñonez, Joanna Ortiz-Alcántara, David Esaú Fragoso-Fonseca, Fabiola Garcés-Ayala, Noé Escobar-Escamilla, Mauricio Vázquez-Pichardo, Alma Núñez-León, María de la Luz Torres-Rodríguez, Belem Torres-Longoria, Irma López-Martínez, Cuitláhuac Ruíz-Matus, Pablo Kuri-Morales, José Ernesto Ramírez-González

**Affiliations:** a Instituto de Diagnóstico y Referencia Epidemiológicos Dr. Manuel Martínez Báez, Mexico City, Mexico; b Facultad de Medicina, UNAM, Mexico City, Mexico; c Dirección General de Epidemiología, Mexico City, Mexico; d Subsecretaría de Prevención y Promoción de la Salud, Mexico City, Mexico

## Abstract

The mosquito-borne chikungunya virus, an alphavirus of the *Togaviridae* family, is responsible for acute polyarthralgia epidemics. Here, we report the complete genome sequences of two chikungunya virus strains, InDRE04 and InDRE51, identified in the Mexican states of Jalisco and Chiapas in 2014. Phylogenetic analysis showed that both strains belong to the Asian genotype.

## GENOME ANNOUNCEMENT

Chikungunya virus (CHIKV) is a mosquito-transmitted alphavirus that causes acute fever and acute and chronic musculoskeletal pain in humans. CHIKV epidemics have been described in Africa, the Middle East, Europe, India, and Southeast Asia (1, 2). Three phylogenetically distinct groups of CHIKV with distinct antigenic properties have been identified, namely, the Asian genotype, the West African genotype, and the East, Central, and Southern African (ECSA) genotype (3, 4). In December 2013, the World Health Organization (WHO) reported the local transmission of chikungunya in St. Martin (5). Since then, this virus has been reported in several countries of the Caribbean and was first detected in the State of Jalisco as the first imported case in Mexico (6, 7). In October 2014, the first autochthonous case was reported in the southeastern Mexican State of Chiapas.

We report here the complete sequences of the genomes of two chikungunya virus strains, InDRE04 (Jalisco) and InDRE51 (Chiapas); InDRE04 was isolated from a 33-year-old woman and identified as an imported case from the Caribbean, and InDRE51 was detected in a 8-year-old girl and identified as the first autochthonous local case in Mexico. Viral RNA of CHIKV was extracted from viral culture in C6/36 cells and directly from serum for InDRE04 and InDRE51, respectively. Four primer pairs were used to generate the amplicons that covered the entire viral genome.

Both strains were sequenced using the 454 FLX-Titanium platform (Roche, Branford, CT). A single-end library was generated for each strain, and 22,384 reads for InDRE04 and 108,396 reads for InDRE51 were obtained. The complete genomes were assembled using Newbler version 2.9, using the sequence of chikungunya virus strain 99659 as a mapping reference (accession no. KJ451624). Two contigs were obtained (2,198 nucleotides [nt] and 9,793 nt), with a total length of 11,991 nt and an average coverage of 499.6× for InDRE04 and only one contig of 11,989 nt with an average coverage of 2995.6× for InDRE51. The contigs were annotated and submitted using the NCBI BankIt tool. Both sequences contain the 5′ and 3′ untranslated regions and two open reading frames (ORFs) of 7,413 nt and 3,747 nt. The first ORF encodes nsP1, nsP2, nsP3, and nsP4 (nonstructural proteins), and the second ORF encodes C, E3, E2, 6K and E1 (structural proteins). An untranslated junction region of 65 nt was observed between the two ORFs.

Preliminary phylogenetic analysis was performed by using MEGA5.0 with the neighbor-joining method; the results demonstrated that both InDRE04 and InDRE51 strains belong to the Asian genotype and are closely related to Chikungunya virus strain 99659, isolated in the British Virgin Islands, presenting 99.89% nucleotide identities. The E1 A226V molecular marker for vector selection was not present in the sequences.

This is the first report of complete genome sequences of CHIKV isolated in Mexico, and detailed sequence and phylogenetic analyses of these genomes will be published elsewhere.

### Nucleotide sequence accession numbers.

The assembled complete genome sequences of the InDRE04 and InDRE51 strains were submitted to GenBank under the accession numbers KP851710 and KP851709, respectively. The versions described in this paper are the first versions.
